# Epidemiology and Impact of *Campylobacter* Infection in Children in 8 Low-Resource Settings: Results From the MAL-ED Study

**DOI:** 10.1093/cid/ciw542

**Published:** 2016-08-07

**Authors:** Caroline Amour, Jean Gratz, Estomih Mduma, Erling Svensen, Elizabeth T. Rogawski, Monica McGrath, Jessica C. Seidman, Benjamin J. J. McCormick, Sanjaya Shrestha, Amidou Samie, Mustafa Mahfuz, Shahida Qureshi, Aneeta Hotwani, Sudhir Babji, Dixner Rengifo Trigoso, Aldo A. M. Lima, Ladaporn Bodhidatta, Pascal Bessong, Tahmeed Ahmed, Sadia Shakoor, Gagandeep Kang, Margaret Kosek, Richard L. Guerrant, Dennis Lang, Michael Gottlieb, Eric R. Houpt, James A. Platts-Mills, for the Etiology, Risk Factors, and Interactions of Enteric Infections and Malnutrition and the Consequences for Child Health and Development Project (MAL-ED) Network Investigators, Angel Mendez Acosta, Rosa Rios de Burga, Cesar Banda Chavez, Julian Torres Flores, Maribel Paredes Olotegui, Silvia Rengifo Pinedo, Mery Siguas Salas, Dixner Rengifo Trigoso, Angel Orbe Vasquez, Imran Ahmed, Didar Alam, Asad Ali, Zulfiqar A. Bhutta, Shahida Qureshi, Muneera Rasheed, Sajid Soofi, Ali Turab, Anita K.M. Zaidi, Ladaporn Bodhidatta, Carl J. Mason, Sudhir Babji, Anuradha Bose, Ajila T. George, Dinesh Hariraju, M. Steffi Jennifer, Sushil John, Shiny Kaki, Gagandeep Kang, Priyadarshani Karunakaran, Beena Koshy, Robin P. Lazarus, Jayaprakash Muliyil, Mohan Venkata Raghava, Sophy Raju, Anup Ramachandran, Rakhi Ramadas, Karthikeyan Ramanujam, Anuradha Rose, Reeba Roshan, Srujan L. Sharma, Shanmuga Sundaram, Rahul J. Thomas, William K. Pan, Ramya Ambikapathi, J. Daniel Carreon, Vivek Charu, Viyada Doan, Jhanelle Graham, Christel Hoest, Stacey Knobler, Dennis R. Lang, Benjamin J.J. McCormick, Monica McGrath, Mark A. Miller, Archana Mohale, Gaurvika Nayyar, Stephanie Psaki, Zeba Rasmussen, Stephanie A. Richard, Jessica C. Seidman, Vivian Wang, Rebecca Blank, Michael Gottlieb, Karen H. Tountas, Caroline Amour, Eliwaza Bayyo, Estomih R. Mduma, Regisiana Mvungi, Rosemary Nshama, John Pascal, Buliga Mujaga Swema, Ladislaus Yarrot, Tahmeed Ahmed, A.M. Shamsir Ahmed, Rashidul Haque, Iqbal Hossain, Munirul Islam, Mustafa Mahfuz, Dinesh Mondal, Fahmida Tofail, Ram Krishna Chandyo, Prakash Sunder Shrestha, Rita Shrestha, Manjeswori Ulak, Aubrey Bauck, Robert Black, Laura Caulfield, William Checkley, Margaret N. Kosek, Gwenyth Lee, Kerry Schulze, Pablo Peñataro Yori, Laura E. Murray-Kolb, A. Catharine Ross, Barbara Schaefer, Suzanne Simons, Laura Pendergast, Cláudia B. Abreu, Hilda Costa, Alessandra Di Moura, José Quirino Filho, Alexandre Havt, Álvaro M. Leite, Aldo A.M. Lima, Noélia L. Lima, Ila F. Lima, Bruna L.L. Maciel, Pedro H.Q.S. Medeiros, Milena Moraes, Francisco S. Mota, Reinaldo B. Oriá, Josiane Quetz, Alberto M. Soares, Rosa M.S. Mota, Crystal L. Patil, Pascal Bessong, Cloupas Mahopo, Angelina Maphula, Emanuel Nyathi, Amidou Samie, Leah Barrett, Rebecca Dillingham, Jean Gratz, Richard L. Guerrant, Eric Houpt, William A. Petri, James Platts-Mills, Rebecca Scharf, Binob Shrestha, Sanjaya Kumar Shrestha, Tor Strand, Erling Svensen

**Affiliations:** 1 Haydom Lutheran Hospital, Haydom, Tanzania; 2 Division of Infectious Diseases and International Health, University of Virginia, Charlottesville; 3 Fogarty International Center, National Institutes of Health; 4 Foundation for the National Institutes of Health, Bethesda; 5 Bloomberg School of Public Health, Johns Hopkins University, Baltimore, Maryland; 6 Haukeland University Hospital, Bergen, Norway; 7 Armed Forces Research Institute of Medical Sciences, Bangkok, Thailand; 8 University of Venda, Thohoyandou, South Africa; 9 International Centre for Diarrhoeal Disease Research, Dhaka, Bangladesh; 10 Aga Khan University, Karachi, Pakistan; 11 Christian Medical College, Vellore, India; 12 Asociación Benéfica PRISMA, Iquitos, Peru; 13 Clinical Research Unit and Institute of Biomedicine, Federal University of Ceara, Fortaleza, Brazil

**Keywords:** *Campylobacter*, children, risk factors, growth, inflammation

## Abstract

***Background.*** Enteropathogen infections have been associated with enteric dysfunction and impaired growth in children in low-resource settings. In a multisite birth cohort study (MAL-ED), we describe the epidemiology and impact of *Campylobacter* infection in the first 2 years of life.

***Methods.*** Children were actively followed up until 24 months of age. Diarrheal and nondiarrheal stool samples were collected and tested by enzyme immunoassay for *Campylobacter*. Stool and blood samples were assayed for markers of intestinal permeability and inflammation.

***Results.*** A total of 1892 children had 7601 diarrheal and 26 267 nondiarrheal stool samples tested for *Campylobacter*. We describe a high prevalence of infection, with most children (n = 1606; 84.9%) having a *Campylobacter*-positive stool sample by 1 year of age. Factors associated with a reduced risk of *Campylobacter* detection included exclusive breastfeeding (risk ratio, 0.57; 95% confidence interval, .47–.67), treatment of drinking water (0.76; 0.70–0.83), access to an improved latrine (0.89; 0.82–0.97), and recent macrolide antibiotic use (0.68; 0.63–0.74). A high *Campylobacter* burden was associated with a lower length-for-age *Z* score at 24 months (−1.82; 95% confidence interval, −1.94 to −1.70) compared with a low burden (−1.49; −1.60 to −1.38). This association was robust to confounders and consistent across sites. *Campylobacter* infection was also associated with increased intestinal permeability and intestinal and systemic inflammation.

***Conclusions.*** *Campylobacter* was prevalent across diverse settings and associated with growth shortfalls. Promotion of exclusive breastfeeding, drinking water treatment, improved latrines, and targeted antibiotic treatment may reduce the burden of *Campylobacter* infection and improve growth in children in these settings.

## Teaser

In a multisite birth cohort study, we document a high burden of *Campylobacter* infection using enzyme immunoassay, demonstrate an association between *Campylobacter* and linear growth shortfalls and both increased intestinal permeability and intestinal and systemic inflammation, and identify potential interventions.

Enteropathogen infection has been associated with impaired growth in young children in low-resource settings [1–4], which in turn has been associated with long-term sequelae, with significant implications for health and human capital [5–7]. Recent work has implicated *Campylobacter* [2, 3], with the putative mechanism being environmental enteric dysfunction, a condition characterized by altered intestinal function and inflammation [8–10]. In high-resource settings, *Campylobacter* infection is sporadic and associated with exposure to undercooked chicken [11, 12], or less frequently common-source outbreaks, often due to contaminated dairy products [13]. In contrast, in low-resource settings, *Campylobacter* infection is frequently endemic [2, 14]. Although exposure to poultry may be important, identified determinants are varied [11, 15–17].

The Etiology, Risk Factors, and Interactions of Enteric Infections and Malnutrition and the Consequences for Child Health and Development Project (MAL-ED) is a birth cohort study performed at 8 sites in South America, sub-Saharan Africa, and Asia [18]. Notably, MAL-ED used an enzyme immunoassay (EIA) to detect *Campylobacter* in stool samples, which is substantially more sensitive than stool culture [19]. Previous work found *Campylobacter* to have the highest attributable burden of diarrhea in this study [20]. Finally, asymptomatic infection with enteropathogens was strongly associated with linear growth shortfalls, with a uniquely strong association between *Campylobacter* and growth (MAL-ED investigators, manuscript in preparation). In the present work, we aimed to describe the burden and impact of *Campylobacter* and identify potential interventions to reduce infection with this pathogen.

## METHODS

### Study Design and Procedures

The MAL-ED study design and methodology have been described elsewhere [18]. The study was conducted at 8 sites: Dhaka, Bangladesh; Vellore, India; Bhaktapur, Nepal; Naushero Feroze, Pakistan; Venda, South Africa; Haydom, Tanzania; Fortaleza, Brazil; and Loreto, Peru. Children were enrolled from November, 2009 to February, 2012 and followed up through 24 months of age. Monthly anthropometry was performed [21]. Stool samples were collected in the absence of diarrhea at 1–12, 15, 18, 21, and 24 months of age as well as from each diarrhea episode, defined as maternal report of ≥3 loose stools in 24 hours or visible blood in stool and identified through twice-weekly home visits.

Caregivers were surveyed biannually from 6 months of age, including questions about maternal income and education, the home environment, drinking water source and treatment, and the presence of animals. Crowding was defined as >2 persons per room living in the home. An improved latrine and water source were defined following World Health Organization guidelines [22]. Treatment of drinking water was defined as boiling, filtering, or adding bleach. Poor access to water was defined as having a primary drinking water source more than a 10-minute walk from the home. Twice-weekly home surveillance assessed breastfeeding in the prior day as exclusive (no consumption of other food or liquid), partial, or none, identified the introduction of specific foods, and recorded antibiotic use. All sites received ethical approval from their respective governmental, local institutional, and collaborating institutional review boards. Written informed consent was obtained from the parent or guardian of each child.

### Laboratory Testing

The laboratory methods used in the MAL-ED study have been described elsewhere [10, 23]. Pertinently, EIA was performed for *Campylobacter* (ProSpecT) as well as *Giardia* and *Cryptosporidium* (TechLab). Monthly surveillance stool samples were also tested for myeloperoxidase (MPO; measured in nanograms per milliliter), a marker of neutrophil activity in the intestinal mucosa (Alpco); neopterin (NEO; measured in nanomoles per liter), a marker of T-helper cell 1 activity (GenWay Biotech); and α-1-antitrypsin (AAT; measured in milligrams per gram), a marker of intestinal permeability (Biovendor). Blood samples collected at 7, 15 and 24 months were tested for α-1-acid glycoprotein (AGP; measured in milligrams per deciliter), a marker of systemic inflammation.

### Data Analysis

To identify factors associated with *Campylobacter* detection in surveillance stool samples, we used generalized estimating equations to fit a generalized linear model with a first-order autoregressive working correlation matrix and robust variance to account for nonindependence of stool testing within each child. To estimate risk ratios for *Campylobacter* detection, we used Poisson regression as an approximation of log-binomial regression since the log-binomial models did not converge [24]. First, we estimated the association for each factor of interest with *Campylobacter* detection, adjusting for age (using a natural spline with knots at 6, 12, and 18 months), sex, site, and season via the terms sin(2mπ/12)+cos(2mπ/12), where *m* is the month of the year as well as—given possible variation in seasonality between sites—an interaction between these terms and site [25]. Then, based on statistical significance, model fit based on the quasi-likelihood information criterion and an assessment of covariance between individual factors, we fit a multivariable model. We also fit site-specific models, excluding those variables from the multivariable model that did not vary within specific sites. Finally, to further describe any association with recent antibiotic use, we fit 2 multivariable models, adjusted as described above, which included (1) class-specific antibiotic use in the prior month and (2) class-specific use in 15-day windows over the prior 60 days.

For the analysis of linear growth, included individuals were required to have a length-for-age *Z* (LAZ) score at 24 months of age. We excluded children from the Pakistan site, owing to bias noted in a subset of length measurements at this site. To estimate the association between the burden of detection of an individual pathogen and 24-month LAZ score, we calculated the enteropathogen burden for each subject using the surveillance stool samples (namely, samples positive/samples tested). Similar burden indices were calculated for the 0–6, 7–12, and 13–24 month intervals. Persistent infection was defined as detection of *Campylobacter* from all surveillance stool samples tested during a 3-month period. We then fit a multiple linear regression, including enrollment LAZ score, sex, site, and *Campylobacter* burden and further adjusted for possible confounders, including factors associated with *Campylobacter* infection in the multivariable model as well as highly-correlated pathogens. To calculate model-predicted 24-month LAZ scores, we calculated predicted population marginal effects using least-squares means [26]. Overall and site-specific high and low burdens of *Campylobacter* were defined as the 90th and 10th percentile of *Campylobacter* burden, respectively.

To estimate the association between *Campylobacter* detection and fecal markers of intestinal permeability and inflammation collected in surveillance stool samples (MPO, NEO, and AAT), we used generalized estimating equations to fit a generalized linear model, as described previously but using a gaussian distribution. These models adjusted for age, sex and site, as previously described, and we also fit site-specific models. Finally, to describe the association between *Campylobacter* burden and systemic inflammation, we fit multiple linear regression models and calculated predicted population marginal effects using least-squares means, both for the entire cohort and for each site, as described for the analysis of linear growth, but with the mean AGP value for each individual as the response variable instead of 24-month LAZ score. All statistical analysis was performed using R software, version 3.2.2 (Foundation for Statistical Computing).

## RESULTS

**Figure 1.**
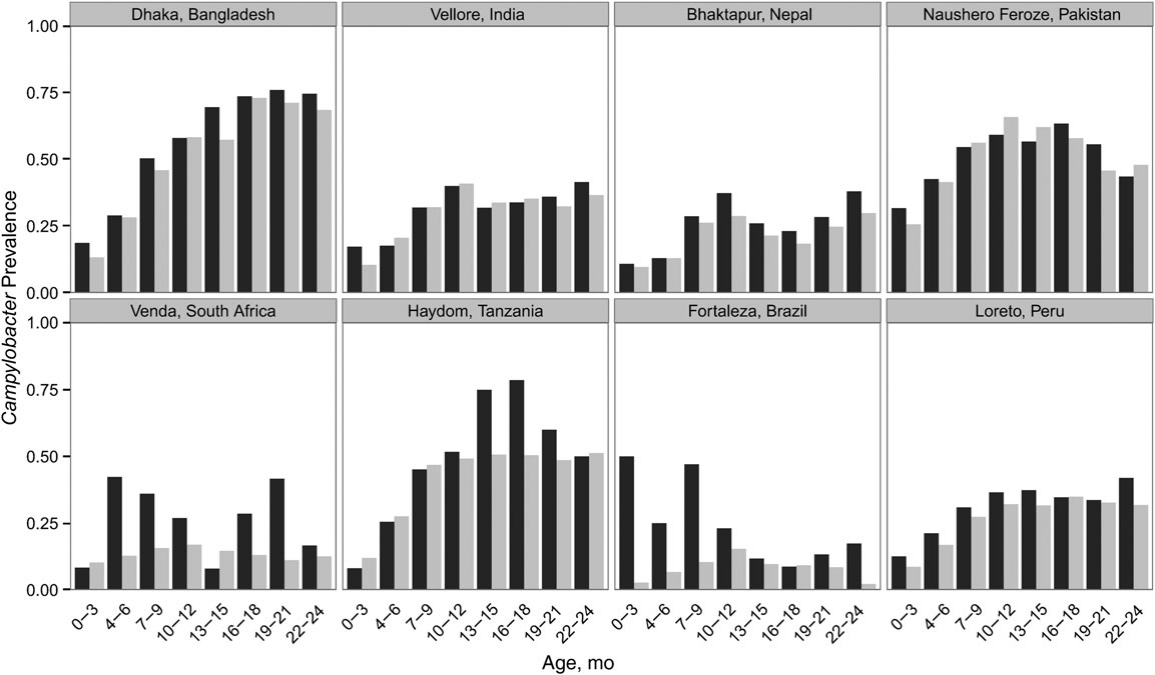
*Campylobacter* prevalence in diarrheal and nondiarrheal surveillance stool samples. Histogram shows proportions of diarrheal (*black*) and surveillance (*gray*) stool samples positive for *Campylobacter* by enzyme immunoassay by age at each site.

A total of 2145 children were enrolled, of whom 2087 had ≥1 surveillance stool sample tested for *Campylobacter*, and 1892 additionally had ≥1 biannual survey performed. These 1892 children had 7601 diarrheal and 26267 surveillance stool samples tested by *Campylobacter* EIA (Supplementary Table 1). *Campylobacter* prevalence in diarrheal and surveillance stool samples varied substantially by site (Figure 1). Generally, *Campylobacter* was frequently detected in surveillance stool samples, with an increasing prevalence over the first year of life and with peak prevalence varying by site, ranging from approximately 10% of surveillance stool samples in Brazil and South Africa to more than half in Bangladesh, Pakistan, and Tanzania. Approximately half of the children had a *Campylobacter-*positive stool sample by 6 months of age, and most (n = 1606; 84.9%) had a *Campylobacter*-positive stool sample by 1 year of age (Supplementary Figure 1). For some sites, exposure was much earlier (eg, in Pakistan, the majority of children had a *Campylobacter*-positive stool sample by 3 months of age). The median proportion of surveillance stool samples positive for *Campylobacter* from 0–24 months was 0.20 (interquartile range, 0.11–0.38). 

**Figure 2.**
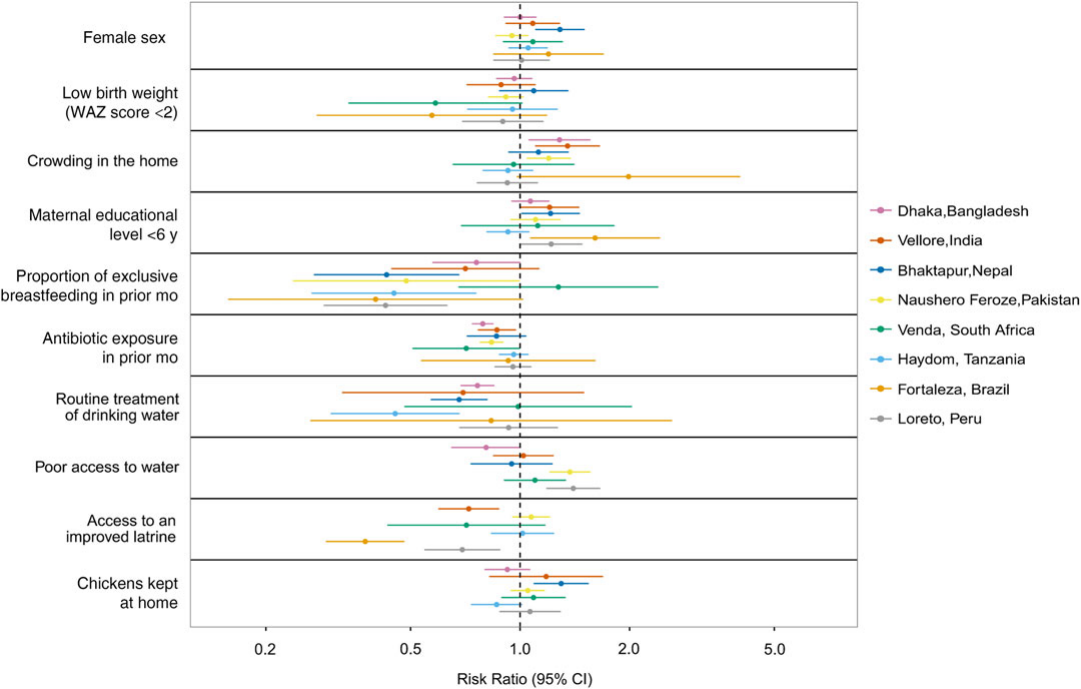
Site-level sensitivity analysis of risk factors for *Campylobacter* detection in surveillance stool samples. Risk ratios with 95% confidence intervals (CIs) are shown for risk factors of interest as identified in the overall model (Table 1). All estimates are adjusted for age, sex, season, and the factors shown in the figure. Factors that did not vary at each site were excluded. Abbreviation: WAZ score, weight-for-age *Z* score.

Possible sociodemographic, environmental, and behavioral determinants of *Campylobacter* infection varied substantially between sites, including crowding in the home (4.7%–83.2%), routine treatment of drinking water (0%–56.5%), and access to an improved latrine (15.1%–100%) (Supplementary Table 1). Overall, the duration of exclusive breastfeeding was short (median, 3.2 months; interquartile range, 2.0–5.3 months) (Supplementary Table 1), and fewer than half of children were exclusively breastfed to 6 months [27]. Nonexclusive breastfeeding generally continued beyond 12 months, with variable cessation in the second year. We identified factors associated with *Campylobacter* detection in surveillance stool samples across all sites (Table 1) and for individual sites (Figure 2). Despite substantial heterogeneity between sites, exclusive breastfeeding in the prior month, routine treatment of drinking water, and access to an improved latrine were most consistently associated with a reduced risk of *Campylobacter* detection. Keeping chickens was marginally associated with increased *Campylobacter* detection, and consumption of chicken was not common in these children (data not shown). 

**Table 1.** Risk Factors for *Campylobacter* Detection in Surveillance Stool Samples

Risk Factors by Category	Risk Ratio (95% CI)	
	Univariable Analysis^a^	Multivariable Analysis^b^
Sociodemographic/maternal		
Female sex	1.06 (1.01–1.12)	1.05 (1.00–1.11)
Low birth weight (WAZ score <2)	0.95 (.89–1.03)	0.93 (.87–1.00)
Crowding in the home	1.19 (1.11–1.27)	1.13 (1.05–1.21)
Maternal educational level <6 y	1.16 (1.09–1.24)	1.08 (1.01–1.15)
Monthly income <$150	1.14 (1.07–1.22)	1.04 (.97–1.11)
Breastfeeding/diet		
Proportion of breastfeeding in prior mo		
Exclusive	0.54 (.46–.62)	0.56 (.47–.67)
Nonexclusive	1.22 (1.14–1.30)	1.02 (.95–1.10)
Consumption in prior mo		
Water	1.24 (1.13–1.36)	1.06 (.96–1.17)
Animal milk	1.06 (1.01–1.12)	…
Solid food	1.21 (1.09–1.34)	…
Child observed to eat nonfood items	1.08 (1.01–1.16)	…
Antibiotics in prior mo	0.86 (.83–.89)	0.86 (.83–.90)
Water		
Routine treatment of drinking water	0.72 (.66–.78)	0.75 (.69–.82)
Improved source of drinking water	0.93 (.82–1.04)	0.96 (.85–1.07)
Poor access to water	1.22 (1.10–1.34)	1.16 (1.06–1.28)
Sanitation: access to an improved latrine	0.85 (.78–.92)	0.89 (.81–.97)
Environment		
Dirt floor in home	1.16 (1.07–1.25)	1.04 (.96–1.13)
Chickens kept at home	1.07 (.99–1.15)	1.06 (.99–1.13)
Cattle kept at home	1.04 (.96–1.12)	…
Agricultural land ownership	0.99 (.92–1.07)	…

Abbreviations: CI, confidence interval; WAZ score, weight-for-age *Z* score.

^a^ Adjusted for age, sex, site, and season.

^b^ Adjusted for age, site, season, and all variables included in multivariable model.

**Figure 3.**
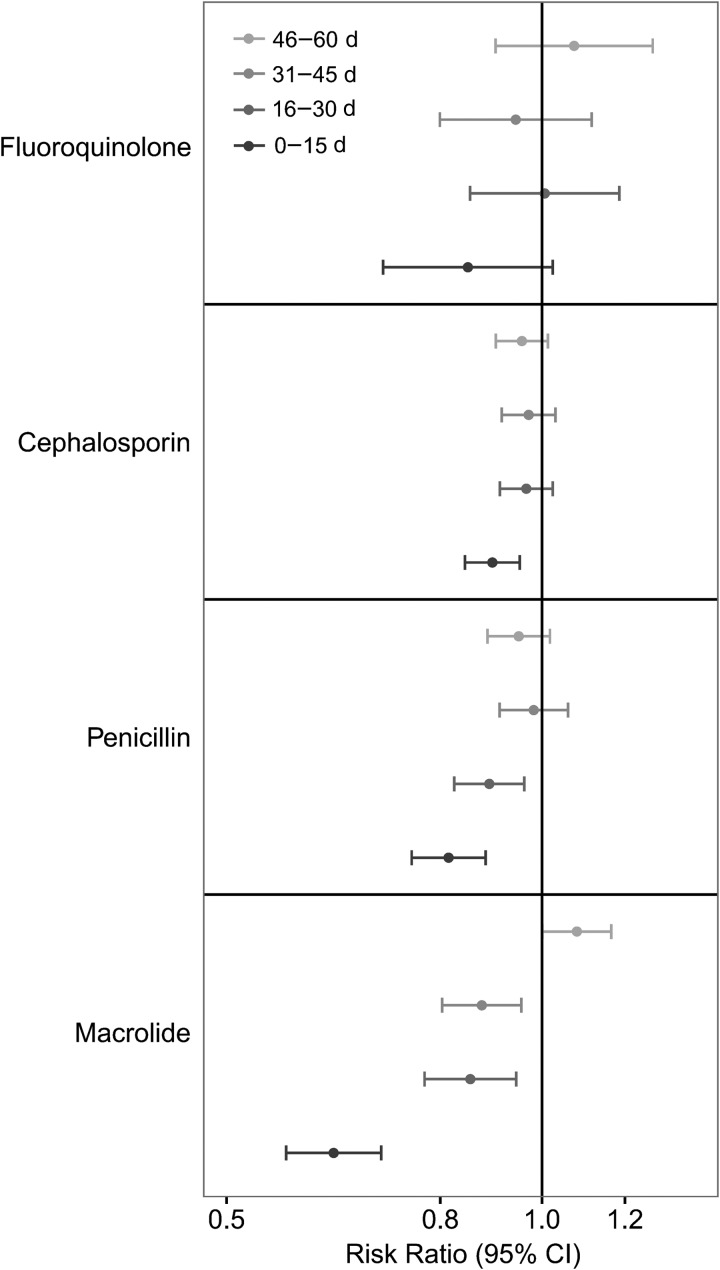
Timing and class of antibiotic use and *Campylobacter* detection in surveillance stool samples. Risk ratios and 95% confidence intervals (CIs) are derived from a single model adjusted for age, sex, site, season, and all shown windows for antibiotic use.

Antibiotic use varied between sites from on average <1 to almost 10 courses of antibiotics per child-year (Supplementary Table 1). Antibiotic use in the prior month was consistently associated with a reduced risk of *Campylobacter* detection (Table 1, Figure 2). Among all classes, macrolide, cephalosporin, penicillin, and fluoroquinolone use were associated with a reduced risk of detection (Table 2), and macrolide use was associated with the most prolonged clearance of *Campylobacter* from stool, with a significant association for macrolide use in the previous 45 days (Figure 3). 

**Table 2.** Association Between Antibiotic Class Administration in the Prior Month and *Campylobacter* Detection in Surveillance Stool Samples

Antibiotic Class	Antibiotic Courses, No.	Risk Ratio (95% CI)
Macrolide	2820	0.68 (.63–.74)
Cephalosporin	3534	0.80 (.76–.85)
Fluoroquinolone	692	0.89 (.78–1.02)
Penicillin	6577	0.92 (.88–.96)
Metronidazole	2289	0.95 (.87–1.02)
Other/unknown	2761	0.95 (.89–1.02)
Sulfonamide	1560	1.02 (.94–1.10)
Tetracycline	42	1.03 (.58–1.83)

Abbreviation: CI, confidence interval.

To examine whether *Campylobacter* detection served as a marker of infection with other enteropathogens, we examined the correlation between the total *Campylobacter* burden from both surveillance and diarrheal stool samples for each individual with that of non-*Campylobacter* bacterial, viral, and parasitic enteropathogens (Supplementary Figure 2). At all sites, *Campylobacter* burden was more strongly correlated with the burden of parasitic enteropathogens (Pearson correlation coefficient, 0.35; *P* < .001) than with non-*Campylobacter* bacterial (0.01; *P* = .55) or viral enteropathogens (0.25; *P* < .001). Protozoal parasites were more strongly associated than helminths (0.35 vs 0.09; both *P* < .001). The majority of protozoal enteropathogen detections were either *Giardia* or *Cryptosporidium*, and *Giardia* correlated most strongly with *Campylobacter* (0.32 vs 0.21; both *P* < .001).

For the 7 sites included in the growth analysis, 1426 of 1892 children had anthropometry performed at enrollment and 24 months. The predicted 24-month LAZ score, adjusted for LAZ score at enrollment and site, was −1.90 (95% confidence interval [CI], −1.99 to −1.80) for children with high *Campylobacter* burden and −1.49 (−1.56 to −1.42) for those with low *Campylobacter* burden. The association persisted after adjustment for potential confounders, specifically sex, crowding in the home, maternal education, monthly income, duration of exclusive breastfeeding, antibiotic use, routine treatment of drinking water, poor access to drinking water, access to an improved latrine, and keeping chickens (high burden, −1.82 [95% CI, −1.94 to −1.70]; low burden, −1.49 [−1.60 to −1.38]).

**Figure 4.**
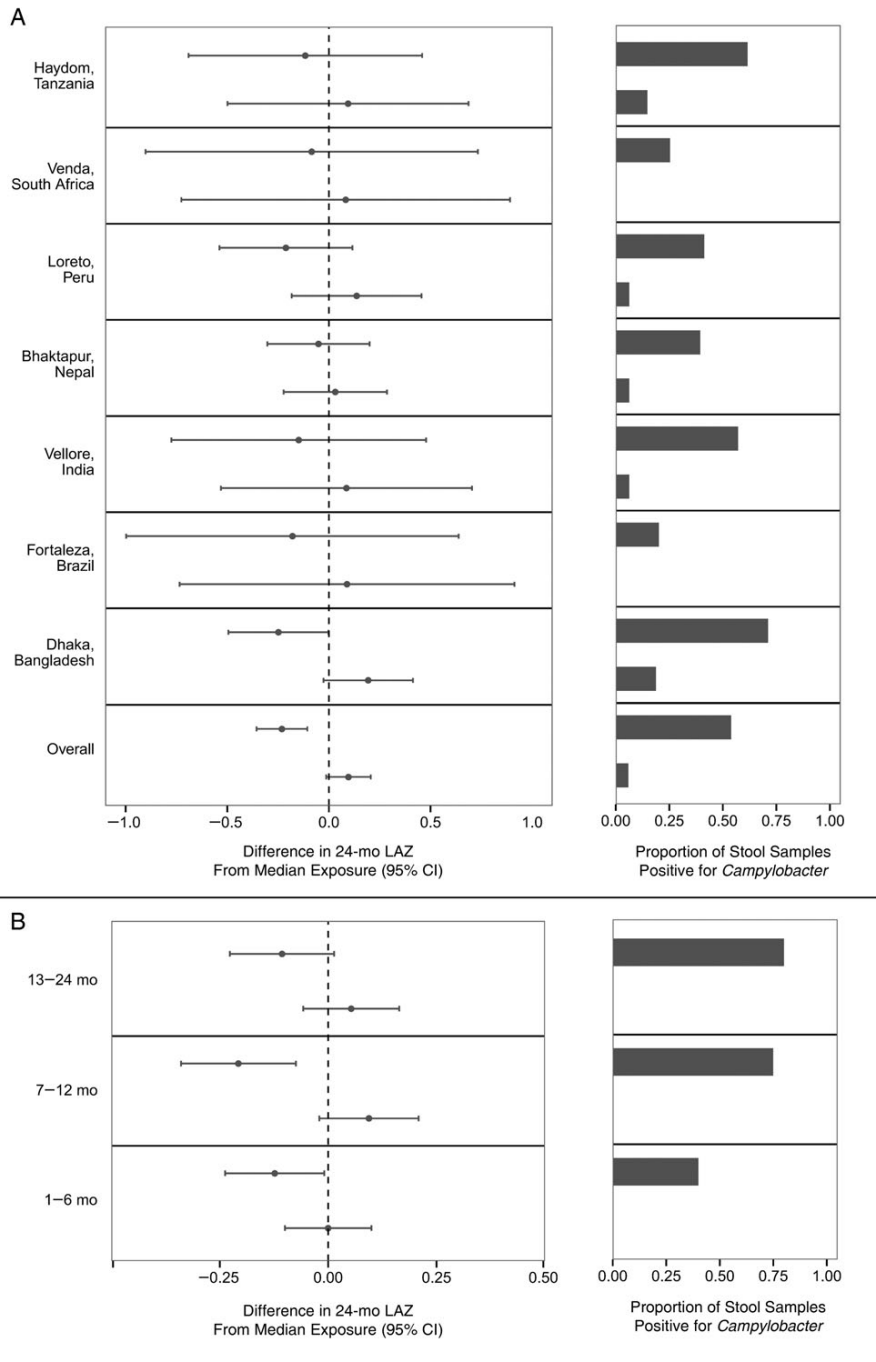
Association between *Campylobacter* burden and length attainment at 24 months. *A*, Left, Difference in model-predicted 24-month length-for-age *Z* (LAZ) score between 10th and 90th percentiles of *Campylobacter* burden and median *Campylobacter* burden, overall and at each site. Right, *Campylobacter* burden expressed as proportion of surveillance stool samples tested that were positive for *Campylobacter. B*, Same estimates for overall 10th and 90th percentiles of *Campylobacter* burden by age interval. Abbreviation: CI, confidence interval.

In site-specific analyses, despite substantial variation in both *Campylobacter* prevalence and associated determinants, the effect was consistent (Figure 4*A*). Additionally adjusting for parasite burden did not substantially change the effect size (0.29 difference in 24-month LAZ score between high and low *Campylobacter* burden after adjustment vs 0.33), and parasite burden was not significantly associated with 24-month LAZ score. We also assessed whether the timing of the *Campylobacter* infection modified the association between *Campylobacter* burden and length attainment, and although the association was seen across all age ranges, it was most notable in children aged 7–12 months (Figure 4*B*). A total of 412 children (28.9%) had ≥1 persistent *Campylobacter* infection during the first year of life. After adjustment for overall burden, having a persistent infection was not associated with 24-month LAZ score (change in 24-month LAZ score, 0.01; 95% CI, −.13 to .15). 

**Figure 5.**
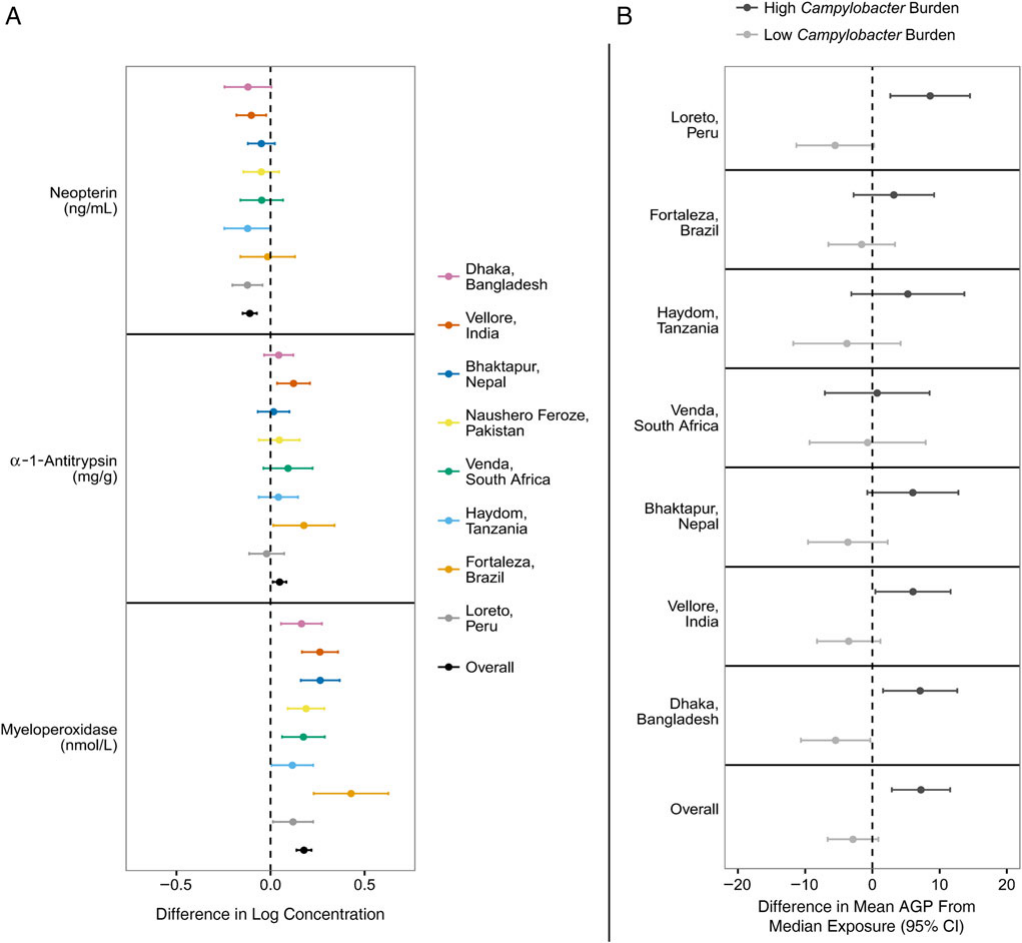
Association between *Campylobacter* detection and fecal markers of intestinal permeability and inflammation (*A*) and systemic inflammation (*B*). *A*, Difference in the log concentrations of neopterin, α-1-antitrypsin, and myeloperoxidase from surveillance stool samples associated with *Campylobacter* detection. *B*, Difference in model-predicted mean α-1-acid glycoprotein (AGP) concentrations between 10th and 90th percentiles of *Campylobacter* burden and median *Campylobacter* burden, both overall and at each site; blood samples were obtained at 7, 15, and 24 months of age. Abbreviation: CI, confidence interval.

We then estimated the association between *Campylobacter* infection and fecal markers of intestinal permeability and inflammation as well as systemic inflammation. *Campylobacter* was detected in ≥1 surveillance stool sample in 1680 of 1892 children (88.8%). With adjustment for age, sex, and site, *Campylobacter*-positive stool samples were associated with higher MPO (difference in log concentration, 0.18 ng/mL; 95% CI, .13–.22 ng/mL) and AAT (0.05 mg/g; 0.02–0.09 mg/g) and lower NEO (−0.12 nmol/L; −0.16 to −0.08 nmol/L) concentrations (Figure 5*A*). The concentration of MPO was higher at the time of the first *Campylobacter* detection (mean [standard deviation], 8.94 [1.24]) than in the month prior (8.86 [1.27]; paired *t* test *P* < .001) or in the month after (8.84 [1.25]; *P* < .001). No such differences were observed for AAT and NEO. Of the 1426 children included in the growth analysis, 1383 had ≥1 blood sample tested for AGP. A high *Campylobacter* burden was associated with a higher mean AGP concentration (difference between high and median burden, 7.1 mg/dL [95% CI, 2.7–11.4]; difference between low and median burden −2.8 [95% CI, −6.6–.9)] (Figure 5*B*). This association was consistent across sites. 

## DISCUSSION

As a prospective, multisite birth cohort study, MAL-ED is the first to use a culture-independent diagnostic for *Campylobacter*, which affords a substantial increase in sensitivity over selective culture [19]. We describe an astonishingly high prevalence of *Campylobacter* infection beginning early in life and a negative association between *Campylobacter* burden and linear growth that is consistent across sites and persists after adjusting for potential confounders. The second half of the first year of life seems the most critical period for *Campylobacter*-associated growth shortfalls, with a large increase in *Campylobacter* prevalence during that time period. These findings build on evidence from the MAL-ED study that subclinical infections with enteropathogens were more strongly linked to linear growth shortfalls than overt diarrhea (MAL-ED investigators, manuscript in preparation).

*Campylobacter* was associated with increased intestinal permeability and local and systemic inflammation, suggesting a mechanism for the association with reduced linear growth [8]. This is consistent with ample evidence that *Campylobacter* can drive intestinal inflammation, in part by altering the composition of the intestinal microbiota, impairing the intestinal barrier, and priming the intestine for chronic inflammatory responses [9, 28–32]. Fecal NEO concentrations were lower in the setting of *Campylobacter* infection, which may support that *Campylobacter* drives inflammation primarily via the innate immune system. A preliminary analysis of a subset of infants aged 0–9 months showed an association between fecal NEO and growth [33], but this association was not seen in the final analysis of the complete cohort.

Despite heterogeneity between sites, factors associated with reduced *Campylobacter* detection included exclusive breastfeeding, treatment of drinking water, access to an improved latrine, and recent use of antibiotics, particularly macrolides. These findings are consistent with other studies which have shown exclusive breastfeeding, water quality, and sanitation to be important determinants of *Campylobacter* infection [34, 35]. These observations, along with the correlation between infection with *Campylobacter* and intestinal parasites, are suggestive of diffuse environmental exposure to *Campylobacter*.

Breastfeeding is thought to reduce the risk of *Campylobacter* infection in infants via passive immunity [36] as well as reducing exposure to the environment, although it has not been shown to improve growth [37]. In the present study, protection was associated with exclusive breastfeeding but not with nonexclusive breastfeeding. However, the observed burden and impact of *Campylobacter* infection extends well beyond the commonly recommended duration of exclusive breastfeeding. The impact of water and sanitation interventions on *Campylobacter* infection has not been evaluated. Clinical trials of water treatment [38] and latrine promotion and construction [39] in India failed to reduce all-cause diarrheal incidence but did not interrogate for specific enteropathogens. A trial of a community-led sanitation program in Mali showed a significant improvement in linear growth [40], an effect that was largest for children <2 years of age, despite no decrease in diarrhea. Intriguingly, the prevalence of bloody diarrhea was reduced, and *Campylobacter* was the primary etiology of bloody diarrhea in infants in the MAL-ED cohorts [20]. Further studies are needed to assess the impact of these interventions on enteropathogen infection and child growth [41].

The observed association between antibiotic use, in particular of the macrolide class, and clearance of *Campylobacter* from surveillance stool samples suggests that antibiotic use could reduce *Campylobacter* burden and possibly improve growth outcomes in these settings. There is evidence from mass treatment trials for trachoma that macrolide administration reduced all-cause mortality, although it is unclear whether undernutrition mediated this effect [42]. Estimates from similar trials that compared the frequency of azithromycin administration for trachoma control have suggested some effect of more frequent azithromycin administration on linear growth [43]. Any observed benefit would have to be weighed against the potential harms of early antibiotic use, in particular selection for drug resistance and collateral effects on the gut microbiota [44–46].

Our work has several limitations. First, Platts-Mills et al [19] have shown that the *Campylobacter* EIA detects a broad range of *Campylobacter* species, including species of nonavian origin and unclear pathogenicity. This may have reduced the association between some risk factors (eg, keeping chickens) and *Campylobacter* infection as well as the association between pathogenic *Campylobacter* species and inflammation and growth. Nucleic acid–based detection may provide a more granular understanding of specific *Campylobacter* species [47]. In the absence of culture isolates and molecular typing, our ability to distinguish persistent infection from reinfection was limited. However, the high prevalence and relatively transient clearance after antibiotic use suggest that reinfection is common. Third, measurement of environmental exposures is difficult, which may preclude the identification of risk factors for *Campylobacter* infection. Finally, it is possible that *Campylobacter* infection is a marker or sequela rather than a cause of reduced linear growth. An assessment of pathogen carriage and growth in a study designed to reduce exposure to *Campylobacter* may better distinguish these 2 possibilities.

In sum, this work provides strong evidence to suggest that *Campylobacter* infection is an important contributor to linear growth shortfalls in children in low-resource settings, given the strength, consistency across diverse settings, and persistence of the observed association with adjustment for potential confounders, the high burden of infection, and the presence of a plausible mechanism. Promotion of exclusive breastfeeding, routine treatment of drinking water, access to improved latrines, and judicious antibiotic administration may reduce *Campylobacter* infection and improve linear growth in children in these settings. We recommend that, in addition to vaccine development, clinical trials be undertaken to reduce *Campylobacter* infections via such interventions, with both subclinical pathogen infection and linear growth as outcomes of interest.

## Supplementary Material

Supplementary Data

Supplementary Data
