# Supplementary material for: Epidemiology and Impact of Campylobacter Infection in Children in 8 Low-Resource Settings: Results From the MAL-ED Study
Source: Clin Infect Dis. 2016 Aug 7;63(9):1171–9. doi: 10.1093/cid/ciw542 (PMC5064165; doi:10.1093/cid/ciw542)

Contents

[MAL-ED Investigators and Institutional Affiliations 2](#_Toc448400088)

[Table S1. Study follow-up and characteristics of study population 3](#_Toc448400089)

[Figure S1. Cumulative incidence of Campylobacter infection by site. 4](#_Toc448400090)

[Figure S2. Relationship between cumulative burden of infection with Campylobacter and non-Campylobacter pathogens. 5](#_Toc448400091)

MAL-ED Investigators and Institutional Affiliations

Angel Mendez Acosta^1^, Rosa Rios de Burga^1^, Cesar Banda Chavez^1^, Julian Torres Flores^1^, Maribel Paredes Olotegui^1^, Silvia Rengifo Pinedo^1^, Mery Siguas Salas^1^, Dixner Rengifo Trigoso^1^, Angel Orbe Vasquez^1^, Imran Ahmed^2^, Didar Alam^2^, Asad Ali^2^, Zulfiqar A Bhutta^2^, Shahida Qureshi^2^, Muneera Rasheed^2^, Sajid Soofi^2^, Ali Turab^2^, Anita KM Zaidi^2,^ Ladaporn Bodhidatta^3^, Carl J Mason^3^, Sudhir Babji^4^, Anuradha Bose^4^, Ajila T George^4^, Dinesh Hariraju^4^, M. Steffi Jennifer^4^, Sushil John^4^, Shiny Kaki^4^, Gagandeep Kang^4^, Priyadarshani Karunakaran^4^, Beena Koshy^4^, Robin P Lazarus^4^, Jayaprakash Muliyil^4^, Mohan Venkata Raghava^4^, Sophy Raju^4^, Anup Ramachandran^4^, Rakhi Ramadas^4^, Karthikeyan Ramanujam^4^, Anuradha Rose^4^, Reeba Roshan^4^, Srujan L Sharma^4^, Shanmuga Sundaram E^4^, Rahul J Thomas^4^, William K Pan^5,6^, Ramya Ambikapathi^6^, J Daniel Carreon^6^, Vivek Charu^6^, Viyada Doan^6^, Jhanelle Graham^6^, Christel Hoest^6^, Stacey Knobler^6^, Dennis R Lang^6, 7^, Benjamin JJ McCormick^6^, Monica McGrath^6^, Mark A Miller^6^, Archana Mohale^6,^ Gaurvika Nayyar^6^, Stephanie Psaki^6^, Zeba Rasmussen^6^, Stephanie A Richard^6^, Jessica C Seidman^6^, Vivian Wang^6^, Rebecca Blank^7^, Michael Gottlieb^7^, Karen H Tountas^7^, Caroline Amour^8^, Eliwaza Bayyo^8^, Estomih R Mduma^8^, Regisiana Mvungi^8^, Rosemary Nshama^8^, John Pascal^8^, Buliga Mujaga Swema^8^, Ladislaus Yarrot^8^, Tahmeed Ahmed^9^, AM Shamsir Ahmed^9^, Rashidul Haque^9^, Iqbal Hossain^9^, Munirul Islam^9^, Mustafa Mahfuz^9^, Dinesh Mondal^9^, Fahmida Tofail^9^, Ram Krishna Chandyo^10^, Prakash Sunder Shrestha^10^, Rita Shrestha^10^, Manjeswori Ulak^10^, Aubrey Bauck^11^, Robert Black^11^, Laura Caulfield^11^, William Checkley^11,6^, Margaret N Kosek^11^, Gwenyth Lee^11^, Kerry Schulze^11^, Pablo Peñataro Yori^11^, Laura E. Murray-Kolb^12^, A Catharine Ross^12^, Barbara Schaefer^12,6^, Suzanne Simons^12^, Laura Pendergast^13^, Cláudia B Abreu^14^, Hilda Costa^14^, Alessandra Di Moura^14^, José Quirino Filho^14,6^, Alexandre Havt^14^, Álvaro M Leite^14^, Aldo AM Lima^14^, Noélia L Lima^14^, Ila F Lima^14^, Bruna LL Maciel^14^, Pedro HQS Medeiros^14^, Milena Moraes^14^, Francisco S Mota^14^, Reinaldo B Oriá^14,^ Josiane Quetz^14^, Alberto M Soares^14^,Rosa MS Mota^14^, Crystal L Patil^16^, Pascal Bessong^17^, Cloupas Mahopo^17^, Angelina Maphula^17^, Emanuel Nyathi^17^, Amidou Samie^17^, Leah Barrett^18^, Rebecca Dillingham^18^, Jean Gratz^18^, Richard L Guerrant^18^, Eric Houpt^18^, William A Petri, Jr^18^, James Platts-Mills^18^, Rebecca Scharf^18^, Binob Shrestha^19^, Sanjaya Kumar Shrestha^19^, Tor Strand^19,15^, Erling Svensen^20,8^

^1^A.B. PRISMA, Iquitos, Peru, ^2^Aga Khan University, Karachi, Pakistan, ^3^Armed Forces Research Institute of Medical Sciences, Bangkok, Thailand, ^4^Christian Medical College, Vellore, India, ^5^Duke University, Durham, NC, USA, ^6^Fogarty International Center/National Institutes of Health, Bethesda, MD, USA, ^7^Foundation for the NIH, Bethesda, MD, USA, ^8^Haydom Lutheran Hospital, Haydom, Tanzania, ^9^icddr,b, Dhaka, Bangladesh, ^10^Institute of Medicine, Tribhuvan University, Kathmandu, Nepal, ^11^Johns Hopkins University, Baltimore, MD, USA, ^12^The Pennsylvania State University, University Park, PA, USA, ^13^Temple University, Philadelphia, PA, USA, ^14^Universidade Federal do Ceara, Fortaleza, Brazil, ^15^University of Bergen, Norway, ^16^University of Illinois at Chicago, IL, USA, ^17^University of Venda, Thohoyandou, South Africa, ^18^University of Virginia, Charlottesville, VA, USA, ^19^Walter Reed/AFRIMS Research Unit, Kathmandu, Nepal, ^20^Haukeland University Hospital, Bergen, Norway

# **Table S1.** Study follow-up and characteristics of study population

|  | Dhaka, Bangladesh | Vellore,  India | Bhaktapur,  Nepal | Naushero Feroze,  Pakistan | Venda, South Africa | Haydom, Tanzania | Fortaleza, Brazil | Loreto,  Peru | Overall |
| --- | --- | --- | --- | --- | --- | --- | --- | --- | --- |
| Number of subjects | 232 | 230 | 233 | 258 | 236 | 239 | 197 | 267 | 1892 |
| Mean duration of follow-up (months) | 22.5 | 23.5 | 23.3 | 23.3 | 23.1 | 21.9 | 21.5 | 21.2 | 22.5 |
| # monthly surveillance stools | 3177 | 3330 | 3444 | 3249 | 3592 | 3377 | 2415 | 3683 | 26267 |
| # diarrheal stools | 1500 | 702 | 922 | 2035 | 159 | 199 | 118 | 1966 | 7601 |
| Female gender | 122 (52.6) | 128 (55.7) | 108 (46.4) | 132 (51.2) | 115 (48.7) | 123 (51.5) | 97 (49.2) | 123 (46.1) | 948 (50.1) |
| Low birth weight  (WAZ < -2) | 50 (21.6) | 49 (21.3) | 31 (13.3) | 65 (25.2) | 15 (6.4) | 7 (2.9) | 10 (5.1) | 25 (9.4) | 252 (13.3) |
| Crowding in the home | 193 (83.2) | 139 (60.4) | 53 (22.7) | 194 (75.2) | 11 (4.7) | 43 (18.0) | 10 (5.1) | 49 (18.4) | 692 (36.6) |
| Maternal education <6 years | 146 (62.9) | 82 (35.7) | 59 (25.3) | 212 (82.2) | 6 (2.5) | 86 (36.0) | 25 (12.7) | 59 (22.1) | 675 (35.7) |
| Monthly income <150 USD | 164 (70.7) | 213 (92.6) | 115 (49.4) | 139 (53.9) | 51 (21.6) | 237 (99.2) | 8 (4.1) | 175 (65.5) | 1102 (58.2) |
| Months of exclusive breastfeeding (Median (IQR)) | 5.4 (4.5 – 6.1) | 4.1 (3.1 – 5.3) | 4.1 (2.5 – 5.4) | 2.0 (2.0 – 2.0) | 2.0 (2.0 – 2.2) | 2.2 (2.0 – 3.5) | 2.9 (2.0 – 4.9) | 6.1 (4.1 – 8.2) | 3.2 (2.0 – 5.3) |
| Months of non-exclusive breastfeeding (Median (IQR)) | 24.0 (21.0 – 24.0) | 17.9 (12.0 – 21.0) | 24.0 (21.0 – 24.0) | 18.0 (12.0 – 23.7) | 18.0 (15.0 – 21.0) | 17.9 (14.1 – 21.0) | 15.0 (8.6 – 24.0) | 17.6 (14.5 – 21.0) | 20.1 (14.7 – 24.0) |
| Child observed to eat  non-food items | 132 (56.9) | 56 (24.3) | 79 (33.9) | 55 (21.3) | 159 (67.4) | 213 (89.1) | 1 (0.5) | 2 (0.7) | 697 (36.8) |
| Courses of antibiotics per child-year | 9.2 | 3.9 | 2.3 | 10.6 | 1.1 | 3.7 | 0.6 | 5.4 | 4.8 |
| Days of antibiotics per child-year | 57.1 | 16.5 | 13.6 | 64.8 | 7.5 | 25.5 | 4.9 | 25.0 | 27.8 |
| Routine treatment of  drinking water | 131 (56.5) | 5 (2.2) | 84 (36.1) | 0 (0) | 8 (3.4) | 4 (1.7) | 6 (3) | 29 (10.9) | 267 (14.1) |
| Improved source of drinking water | 232 (100) | 230 (100) | 233 (100) | 258 (100) | 180 (76.3) | 74 (31) | 197 (100) | 238 (89.1) | 1642 (86.8) |
| Poor access to water | 1 (0.4) | 60 (26.1) | 31 (13.3) | 1 (0.4) | 121 (51.3) | 239 (100) | 0 (0) | 108 (40.4) | 561 (29.7) |
| Access to an improved latrine | 232 (100) | 108 (47) | 233 (100) | 197 (76.4) | 229 (97) | 36 (15.1) | 196 (99.5) | 54 (20.2) | 1285 (67.9) |
| Dirt floor in home | 8 (3.4) | 5 (2.2) | 121 (51.9) | 173 (67.1) | 8 (3.4) | 225 (94.1) | 0 (0) | 178 (66.7) | 718 (37.9) |
| Keep chickens at the home | 3 (1.3) | 11 (4.8) | 62 (26.6) | 102 (39.5) | 70 (29.7) | 219 (91.6) | 0 (0) | 70 (26.2) | 537 (28.4) |
| Keep cattle at the home | 0 (0) | 5 (2.2) | 3 (1.3) | 140 (54.3) | 23 (9.7) | 148 (61.9) | 0 (0) | 0 (0) | 319 (16.9) |
| Own agricultural land | 12 (5.2) | 1 (0.4) | 155 (66.5) | 136 (52.7) | 103 (43.6) | 228 (95.4) | 0 (0) | 5 (1.9) | 640 (33.8) |

WAZ, weight-for-age Z score. USD, United States Dollars.

Figure S1. Cumulative incidence of Campylobacter infection by site.


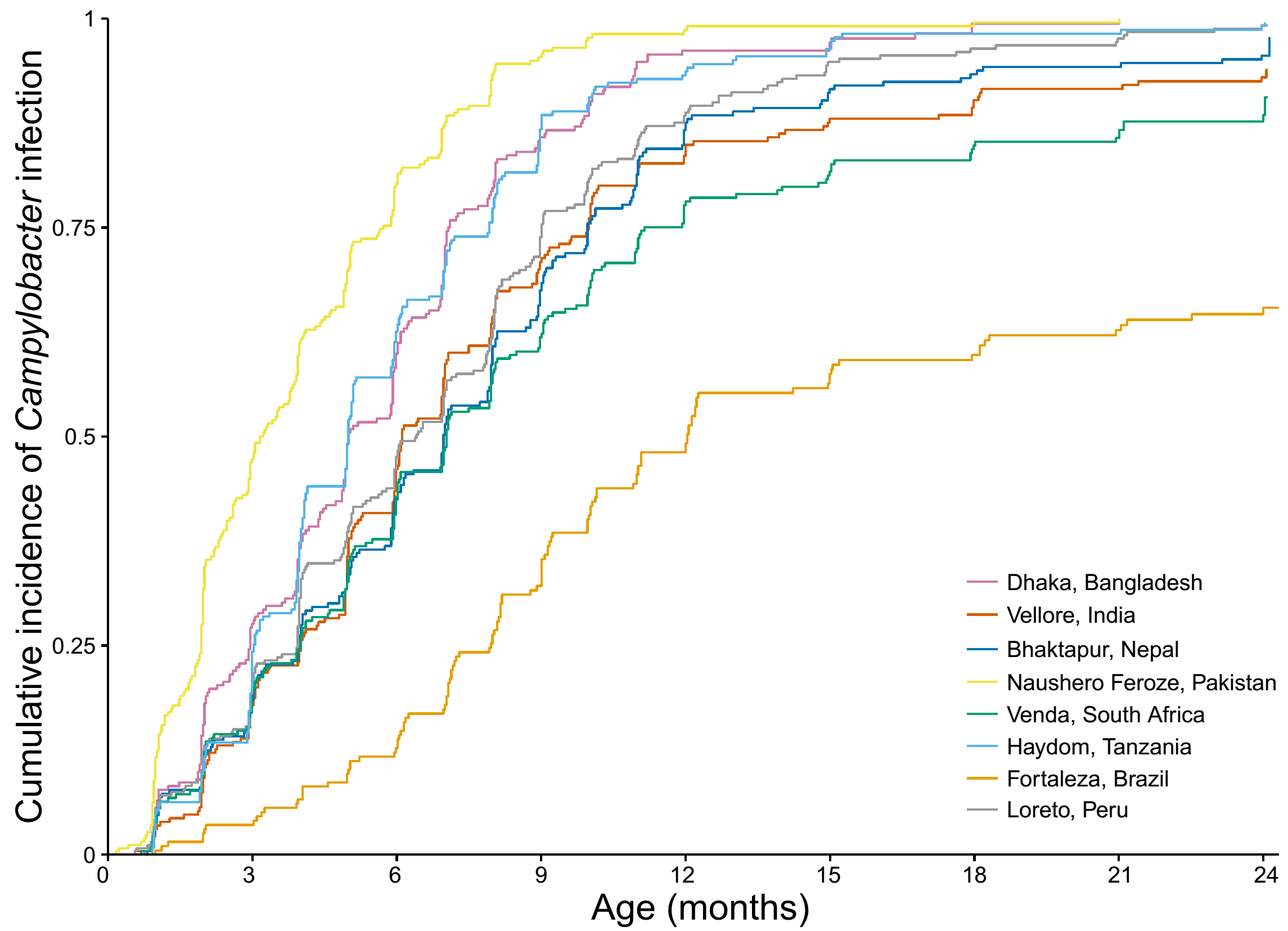


Figure S2. Relationship between cumulative burden of infection with Campylobacter and non-Campylobacter pathogens. In each figure, the x-axis represents the *Campylobacter* pathogen burden index (see Methods), and the y-axis the burden index for bacterial (A), viral (B), and parasitic (C) enteropathogens. Both diarrheal and non-diarrheal stools are included. P values are from Pearson correlation.


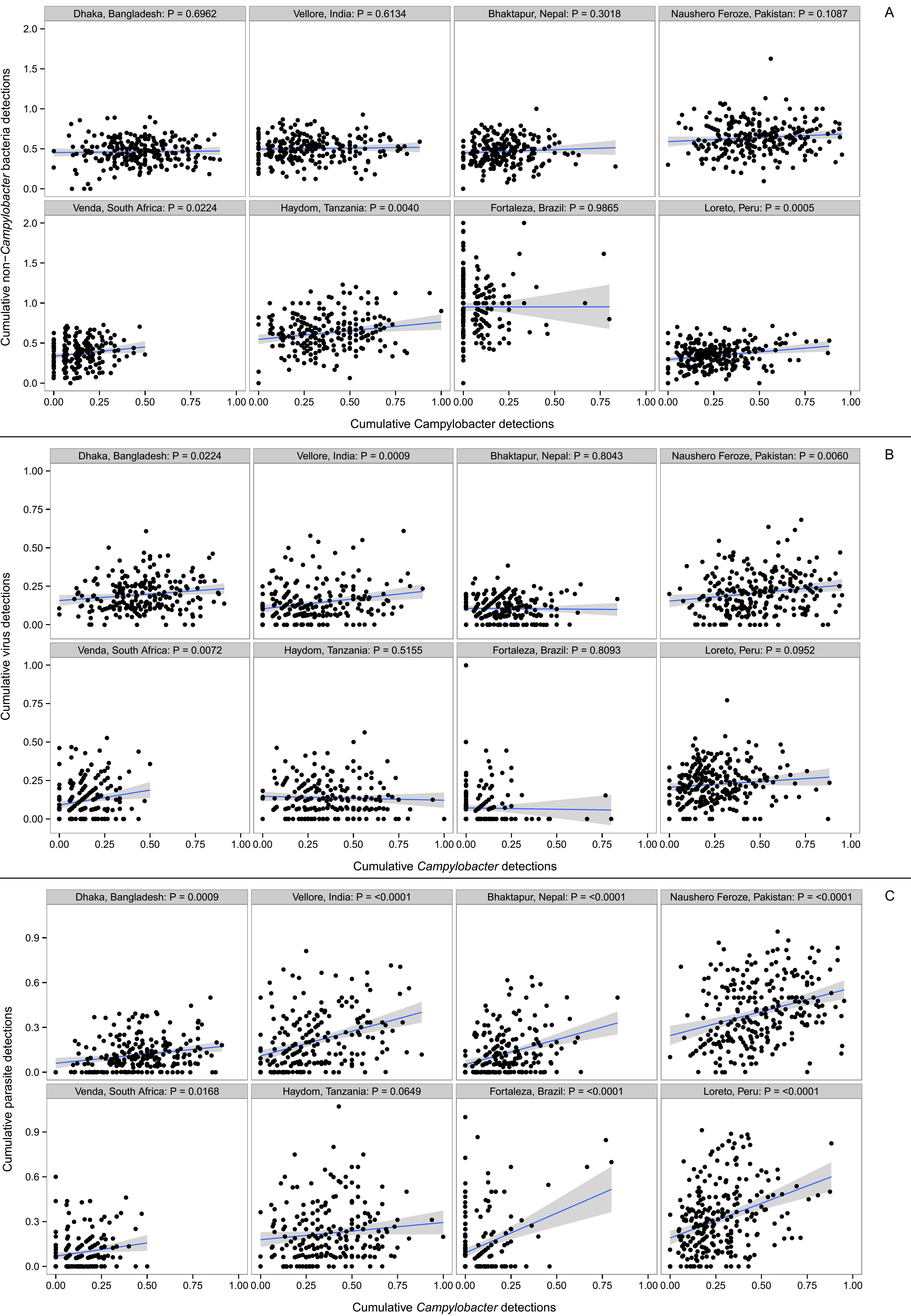

Supplement: Supplementary Data [file ciw542_Supplementary_Data.zip › ciw542supp.docx]
