# Supplementary material for: Epidemiology and Impact of Campylobacter Infection in Children in 8 Low-Resource Settings: Results From the MAL-ED Study
Source: Clin Infect Dis. 2016 Aug 7;63(9):1171–9. doi: 10.1093/cid/ciw542 (PMC5064165; doi:10.1093/cid/ciw542)
Supplement: Supplementary Data [file supp_63_9_1171__index.html]

Epidemiology and impact of Campylobacter infection in children in eight low-resource settings: results from the MAL-ED study — Epidemiology and Impact of Campylobacter Infection in Children in 8 Low-Resource Settings: Results From the MAL-ED Study — Epidemiology and Impact of Campylobacter Infection in Children in 8 Low-Resource Settings: Results From the MAL-ED Study — Supplementary Data 

# Epidemiology and Impact of *Campylobacter* Infection in Children in 8 Low-Resource Settings: Results From the MAL-ED Study

## Supplementary Data

Supplementary Data

- Supplementary Data - Docx file
